# Safety and efficacy of bio-engineered, autologous dermo-epidermal skin grafts in reconstructive surgery: 1-year results of a prospective, randomized, intra-patient controlled, multicenter phase II clinical trial

**DOI:** 10.1177/20417314261429663

**Published:** 2026-03-23

**Authors:** Frederique M. Kemme, Anouk Pijpe, Matthea M. Stoop, Paul P. M. van Zuijlen, Martin Meuli, Clemens Schiestl, Fabienne Hartmann-Fritsch, Bong-Sung Kim, Kathrin Neuhaus, Jan A. Plock, Daniel Rittirsch, Kathi Mujynya, Ernst Reichmann, Sophie Böttcher-Haberzeth, Melinda Farkas, Jenny Bressan, Marcello Zamparelli, Ilaria Mataro, Carlo Petroccione, Alex Pontini, Bruno Azzena, Daniela Marino, Esther Middelkoop

**Affiliations:** 1 Alliance of Dutch Burn Care, Burn Centre, Red Cross Hospital, Beverwijk, The Netherlands; 2 Department of Plastic, Reconstructive and Hand Surgery, Amsterdam UMC location Vrije Universiteit Amsterdam, Netherlands; 3 Amsterdam Movement Sciences, Tissue Function and Regeneration, Amsterdam UMC, The Netherlands; 4 Department of Plastic, Reconstructive and Hand Surgery, Red Cross Hospital, Beverwijk, The Netherlands; 5 Amsterdam UMC Location University of Amsterdam, Pediatric Surgical Centre, Emma Children’s Hospital, The Netherlands; 6 University of Zurich, Switzerland; 7 CUTISS AG, Schlieren, Switzerland; 8 Pediatric Burn Center, Children’s Skin Center, Department of Surgery, University, Children’s Hospital Zurich, University of Zurich, Switzerland; 9 Department of Surgery, University Children’s Hospital Zurich, University of Zurich, Switzerland; 10 Children’s Research Center, University Children’s Hospital Zurich, University of Zurich, Switzerland; 11 Department of Plastic Surgery and Hand Surgery, Cantonal Hospital Aarau, Switzerland; 12 Department of Plastic Surgery and Hand Surgery, Burn Center, University Hospital Zurich, Switzerland; 13 Tissue Biology Research Unit, Department of Surgery, University Children’s Hospital Zurich, University of Zurich, Schlieren, Switzerland; 14 Plastic Surgery and Burns Unit, Santobono Pausilipon National Children’s Hospital, Napoli, Italy; 15 Department of Plastic Reconstructive Surgery and BURNS, AORN A. Cardarelli, Napoli, Italy; 16 U.O.C Grandi Ustionati Azienda Ospedale University of Padova, Italy

**Keywords:** bioengineered skin, tissue engineering, scar revision, scar quality, reconstructive surgery

## Abstract

Extensive full-thickness defects remain challenging to reconstruct, as various techniques used, such as split-thickness skin grafts, often result in donor site morbidity and functional and cosmetic scar sequelae. This study evaluated the safety and efficacy of denovoSkin™—a bio-engineered autologous dermo-epidermal skin graft consisting of patient-derived epidermal and dermal cells cultured within an extracellular matrix—compared to autologous split thickness skin grafts (STSG). Twenty-three patients (mean age 37.4 years; 65% male; 70% post-burn scars), received both denovoSkin™ and STSG on comparable wound areas. At 3 months, scar quality was significantly better for denovoSkin™ than for STSG (POSAS observer total score 23.4 vs 27.9; *p* = 0.008), with benefits maintained at 12 months. Elasticity parameters also consistently favored denovoSkin™, and the ratio of covered surface area to donor site was markedly higher (8.5 vs 0.9; *p* < 0.001). DenovoSkin™ is a safe and effective treatment for full-thickness skin defects, providing favorable long-term skin quality. **Clinical Trial Registration:** Clinicaltrials.gov NCT03394612.

## Introduction

Extensive skin defects and scars from burns, trauma, congenital or acquired diseases, or former surgical procedures (like flap donor sites) pose a major reconstructive challenge. Common reconstructive techniques include split thickness skin grafts (STSG), full thickness skin grafts (FTSG), dermal templates, and various types of flaps. Each method comes with its own challenges: while STSGs are effective for epithelial coverage, healing may be accompanied by contractures and hypertrophic scarring, and donor sites may induce morbidity.^1–3^ Combining STSG with dermal templates may improve outcomes, though considerable scarring remains common. FTSGs and flaps can offer better outcomes but are limited by the availability and size of suitable donor tissue.

In recent decades, alternatives have been introduced such as cultured epithelial autografts (CEAs), whose use is limited by inconsistent graft take, mechanical fragility, and a tendency toward hypertrophic scarring.^4–6^ Next in line, dermal templates were developed to improve the outcome of deep dermal and full thickness wounds in terms of scarring.^7–10^ Advances in tissue engineering have enabled the development of dermo-epidermal skin grafts, containing both keratinocytes and dermal fibroblasts to better mimic full-thickness skin.^11–17^ Among these, denovoSkin™—an autologous, bi-layered engineered human skin graft with keratinocytes and fibroblasts—has shown promise in preclinical studies and early clinical applications.^18,19^ DenovoSkin™ is generated from patient’s own dermal fibroblasts and epidermal keratinocytes, cultured and expanded in vitro within an extracellular matrix (collagen hydrogel) to form a bilayer skin construct.

A phase I first-in-man open prospective, clinical trial in 2014 confirmed safety of denovoSkin™ in both pediatric and adult patients,^20,21^ and a phase II clinical trial in acute burn patients is presently ongoing (NCT03229564, NCT03227146; *manuscript submitted (SB, personal communication*). However, comparative data for reconstructive patients are not yet available, nor are data on long-term scar quality outcomes for reconstructive indications. Here, we report on a prospective, randomized, intra-patient controlled multicenter phase II study in reconstructive patients.

## Methods

### Study design

This phase II study was conducted to evaluate the efficacy and safety of an autologous bio-engineered dermo-epidermal skin graft, denovoSkin™, in comparison to autologous STSGs in patients undergoing planned elective scar reconstruction (trial ID NCT03394612). Patients were enrolled in three study sites in Italy (Santobono Pausilipon National Children’s Hospital, Napoli, AORN A. Cardarelli, Napoli and U.O.C Grandi Ustionati Azienda Ospedale University of Padova, Italy) two sites in The Netherlands (Red Cross Hospital, Beverwijk and Amsterdam University Medical Centers, location Vrije Universiteit, Amsterdam), and two sites in Switzerland (University Children’s Hospital Zurich, and University Hospital Zurich, Zurich, Switzerland). The study protocol closely aligned with the protocol of a previously approved clinical trial investigating denovoSkin™ in burn patients (trial ID NCT0322714, NCT03229564). The study protocol was approved by the Dutch Central Committee on Research Involving Human Subjects (CCMO; NL64565.000.18) and conducted in compliance with the ethical principles of the Declaration of Helsinki (2013), as well as the Good Clinical Practice (GCP) guidelines (ICH E6), the International Council for Harmonization of Technical Requirements for Pharmaceuticals for Human Use (ICH), and any applicable local laws and regulations. Ethics committees and local authorities approved the study protocol. Written informed consent was obtained from each patient or their legally authorized representative before any study-related activities.

### Participants

Eligible participants were patients (⩾1 years old) with full-thickness defects that required coverage in planned elective procedures. Inclusion and exclusion criteria are summarized in Table 1. The minimum wound area required was stratified by age category as follows: 9 (1–5 years), 25 (6–16 years), and 45 cm^2^ (>16 years). A sample size of 20 patients was calculated to provide 80% power to detect a statistically significant difference at a 5% significance level. Considering possible dropouts prior to assessment of the primary endpoint, this number was increased to 25.

**Table 1.** Inclusion and exclusion criteria.

Inclusion criteria
Age: ⩾1 year of age
Large full-thickness defects that require coverage after excision of: • Scars • Benign skin tumors (e.g. neurofibroma) • Melanocytic nevus (e.g. giant nevus) • Gender reassignment surgery • Soft tissue defect after trauma • Soft tissue defect after infection and debridement (e.g. necrotizing fasciitis, hidradenitis suppurativa, purpura fulminans) • Flap donor site (e.g. radial forearm flap)
Minimal areas requiring coverage (not counting the head and neck area for study patients in The Netherlands): • Minimum: 1–5 years: 9 cm^2^ • Minimum: 6–16 years: 25 cm^2^ • Minimum: >16 years: 45 cm^2^
Signed informed consent from the patient or the parents/legally authorized representative.
Exclusion criteria
Tested positive for HBV, HCV, syphilis or HIV
Known underlying or concomitant medical conditions that may interfere with normal wound healing (e.g. systemic skin and connective tissue diseases, any kind of congenital defect of metabolism including insulin-dependent diabetes mellitus, Cushing syndrome or disease, scurvy, chronic hypothyroidism, congenital or acquired immunosuppressive condition, chronic renal failure, or chronic hepatic dysfunction (Child-Pugh class B or C), severe malnutrition, or other concomitant illness which, in the opinion of the Investigator, has the potential to significantly delay wound healing)
Severe drug and alcohol abuse
Pre-existing coagulation disorders as defined by INR outside its normal value, PTT > ULN and fibrinogen < LLN prior to the current hospital admission and / or at the Investigator’s discretion
Known allergies to amphotericin B, gentamicin, penicillin, streptomycin, or bovine collagen
Previous enrollment of the patient into the current phase II study
Participation of the patient in another study with conflicting endpoints within 30 days preceding and during the present study
Patient or parents/legally authorized representative expected not to comply with the study protocol (including patients with severe cognitive dysfunction/impairment and severe psychiatric disorders)
Pregnant or breastfeeding females
Intention to become pregnant during the clinical course of the study (12 months)
Wounds in the head and neck area as study target area (only applicable for study patients in The Netherlands)
Enrollment of the Investigator, his/her family members, employees, and other dependent persons

### Study procedures

#### Production of denovoSkin^TM^

A split-thickness skin biopsy of approximately 4 cm^2^ was harvested from the patient requiring coverage of a full thickness defect, with an electric dermatome set at 0.2 mm. Autologous keratinocytes and fibroblasts were isolated and expanded in vitro. Subsequently, fibroblasts were incorporated into a bovine type I collagen hydrogel (Symatese, Chaponost, France), compressed and after a culture phase, keratinocytes were seeded on top to create the bi-layered dermo-epidermal skin graft denovoSkin™ .^21^ The development and preclinical data on this construct were published earlier.^11,12,14,22^ After a production time of approximately 30 days, one or two pieces of denovoSkin™ (size 45 ± 4 cm^2^, thickness 0.5–2.0 mm) were shipped to the study sites in temperature-controlled conditions under Good Distribution Practice. Until February 2020, denovoSkin™ was manufactured in the GMP-certified clean room facility of Wyss Zurich, Zurich, Switzerland, and thereafter in the GMP-certified clean room facility of CUTISS AG, Schlieren, Switzerland. The production process is operating under GMP regulations involving in process controls, process validations and product release specifications. These include: graft’s integrity, geometry & thickness, cell count in graft, cells viability, histology and sterility.

#### Transplantation and follow-up

For each patient, two wound areas, each of maximal 90 cm^2^, were randomly allocated 1:1 to treatment with either denovoSkin™ or STSG. Randomization of study area treatment assignments was performed using software-based block randomization without stratification and documented in the electronic case report form (eCRF). To avoid bias in wound bed preparation procedures, randomization took place after completion of wound bed preparation. Skin defects were either excised (in case of tumor resection or flap donor site revision) or incised (in case of scar contractures). The wound bed was grafted with 1 or 2 grafts of denovoSkin™ (experimental area) and unmeshed or meshed up to 1:2 STSG (control area), taken with an electric dermatome set at 0.2 mm. The decision whether to mesh was left to the treating surgeon and based on clinical judgment of the wound bed, such as limited vascularization or anticipated exudation.

After transplantation, both study areas were dressed with Mepilex (Ag). Graft take of both study areas was assessed by an experienced clinician during the first dressing change, 6–10 days after grafting, and subsequently on day 21 and 28 after grafting. Throughout the wound healing process, both areas were treated similarly. Scar therapy mainly consisted of pressure garments and/or silicone therapy. Patients returned for follow-up visits at 2, 3, 6, and 12 months after grafting. At 3, 6, and 12 months, the Cutometer (MPA580, Courage Khazaka) was used to assess skin elasticity, the DSM II Colormeter (Cortex Technology) to assess skin color (erythema and melanin), and scar quality was assessed using the Patient and Observer Scar Assessment Scale (POSAS) version 2.0.^23^ Photographic documentation of the study areas was performed at each visit. Adverse events, clinical laboratory results, and clinical and microbiological signs of infection were reported for safety assessment.

### Study endpoints

#### Safety evaluation

Safety evaluation included signs of infection (6–10 days and 21 days post-grafting, assessed by a combination of clinical symptoms and bacterial culture results), adverse events for the full duration of the study, laboratory results, vital signs, and physical examination.

#### Primary efficacy endpoint

The primary endpoint was the scar quality of the study areas based on the POSAS observer scale total score (sum of items excluding overall opinion score), 3 months post-grafting. Higher total POSAS scores indicate worse scar quality, which is measured relative to the patient’s healthy skin.

#### Secondary efficacy endpoints

Secondary efficacy endpoints were wound healing characteristics (graft take, time to wound closure (⩾95% epithelialization)), scar elasticity parameters (Cutometer), scar color parameters (Colormeter), and POSAS patient- and observer scale items.

### Statistical analysis

Continuous variables were summarized using descriptive statistics, while categorical variables were presented through frequency distributions. The difference in the primary efficacy endpoint (POSAS observer total score at 3 months) between the experimental and control area was analyzed by the two-sided paired *t*-test . For other outcomes assessed in both study areas, differences were similarly analyzed using two-sided paired *t*-tests. For Cutometer analyses, measurements with an R0 value of zero were excluded due to unreliability of the measurement. A p-value of <0.05 was considered statistically significant.

## Results

### Patient demographics

**Figure 1.**
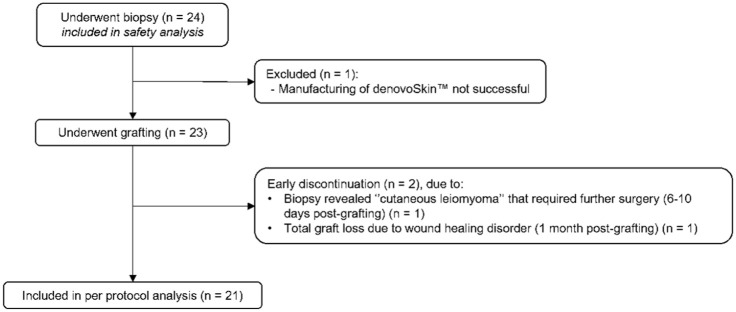
Flowchart of study participants.

Between February 2018 and September 2024, a total of 24 patients underwent biopsy for denovoSkin™ manufacturing, and 23 patients proceeded to transplantation and were included in the final analysis (Figure 1). For one subject, the product could not be manufactured and the subject had to be withdrawn. Two patients dropped out after the grafting procedure; data collected up to their respective follow-ups was included in the analysis. Patient demographics and treatment characteristics are shown in Table 2. Of these patients, 16 (69.6%) were transplanted due to scar revision of a previous burn. Their mean initial total body surface area (TBSA) was 34.4% (SD 26.2%).

**Table 2.** Demographics and treatment characteristics in 23 patients.

Patient characteristics	*N* = 23	
Gender, *N* (%)		
Male	15 (65.2%)	
Female	8 (34.8%)	
Age, mean (SD, min–max)	37.4 (17.7, 3–67)	
Etiology of defect, *N* (%)		
Post-burn scars	16 (69.6%)	
Scar excision (not related to a burn injury)	6 (26.1%)	
Excision of benign skin tumor	1 (4.3%)	
Treatment characteristics	denovoSkin™	STSG
Size (cm^2^) harvested area, mean	4.88	62.7
Size (cm^2^) covered area, mean	52.1	62.9
Expansion ratio, mean (SD)	8.5 (4.4)	0.9 (0.22)
Days to wound closure (⩾95% epithelialization), median	60.5	22.0

**Figure 2.**
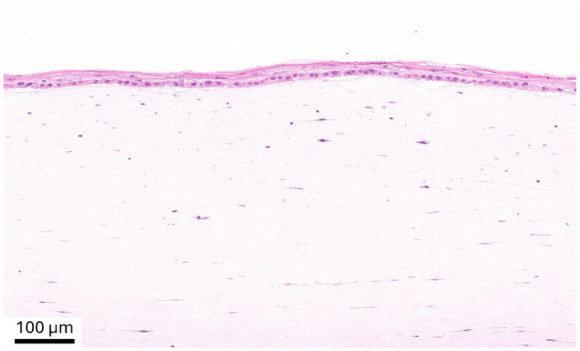
Histological analysis (H&E staining) of the construct denovoSkin^™^ prior to transplantation.

The mean size of the biopsy for denovoSkin™ was 4.88 cm^2^, and the mean split-thickness skin donor area was 62.7 cm^2^. In most cases (20 out of 23, 87%), the control area was treated with an unmeshed STSG. In three patients (13%), a meshed STSG was used (expansion ratio 1:1.5 in two patients, 1:2 in one patient). The mean expansion ratio for denovoSkin™ was 8.5 (SD 4.4), and for STSG 0.9 (SD 0.22; *p* < 0.001). An example of the histological analysis (H&E staining) of a denovoSkin™ product at release is provided in Figure 2, showing the full construct dermis and epidermis.

### Wound healing

**Figure 3.**
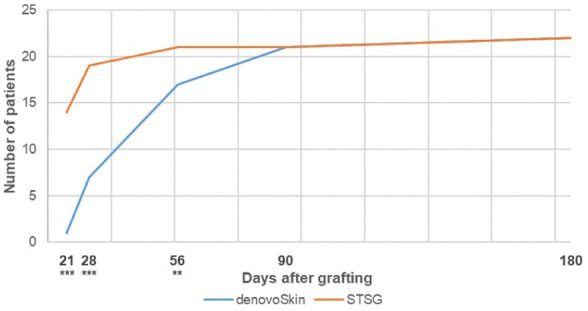
Number of patients with complete wound closure (⩾95% epithelialization) over time for denovoSkin™ (*N* = 22) and STSG (*N* = 22). Symbols indicate statistically significant differences between denovoSkin^™^ and STSG. **p < 0.01. ***p < 0.001.

Graft take was assessed between postoperative days 6 and 10 and was 69.8% (SD 31.8) in the denovoSkin™ area, compared to 85.0% (SD 14.5) in the STSG area (*p* = 0.053). Wound closure over time for denovoSkin™ areas and STSG is shown in Figure 3. Wounds were closed for two-thirds of patients after 56 days for denovoSkin™, and after 21 days for STSG. The difference in wound closure was statistically significant between treatment groups at 21, 28, and 56 days.

### Safety evaluation

A safety evaluation was performed on data of 24 patients that underwent biopsy. An overview of (serious) adverse events that occurred during the study period is presented in Table 3. Over the total safety period, defined as the time between biopsy and end of the study (12 months post transplantation), a total of 57 events were reported for 17 patients, of which 18 events in 9 patients were serious. The most frequently reported adverse events were staphylococcal skin infections (29%) and scar contraction (17%). Infections in study areas occurred in six patients, two of these were in denovoSkin™ treated areas only (*p* = 0.157). Five patients (20.8%) experienced a total of seven events that were possibly related to denovoSkin™, which included infections (8.3%), excessive granulation tissue (4.2%), pruritus (4.2%), impaired healing (4.2%), and traumatic ulcer (4.2%). One of these events (impaired healing requiring additional surgery) was classified as serious, and two events in two patients (soft tissue neoplasm and impaired healing) lead to study discontinuation (after 1 week and after 1 month). No deaths were reported.

**Table 3.** Overview of all (serious) adverse events reported during total safety period in 24 patients, including seriousness and possible relation to denovoSkin^™^.

	Adverse events			Serious adverse events^b^		
Safety analysis	*n* (%)	nae	Relationship to dS (nae)	*n* (%)	nae	Relationship to dS (nae)
**Any**	**8 (33.3%)**	**39**	**5 (20.8%)**	**9 (37.5%)**	**18**	**1 (4.2%)**
Infections and infestations						
Staphylococcal skin infection	7 (29.4%)	7	Possibly (1), unlikely (1), unrelated (5)			
Urinary tract infection bacterial	2 (8.3%)	2	Unrelated			
Wound infection bacterial	2 (8.3%)	2	Probably (1)			
Enterococcal infection	1 (4.2%)	1	Possibly (1)			
Erysipelas				1 (4.2%)	1	Unrelated
Lymphangitis				1 (4.2%)	1	Unrelated
Paronychia	1 (4.2%)	1	Unrelated			
Subcutaneous abscess	1 (4.2%)	1	Unrelated			
Injury and procedural complications						
Scar contraction				4 (16.7%)	7	Unrelated
Post procedural hematoma				1 (4.2%)	1	Unrelated
Contusion	1 (4.2%)	1	Unrelated			
Graft loss	1 (4.2%)	1	Unrelated			
Iatrogenic injury	1 (4.2%)	1	Unrelated			
Seroma	1 (4.2%)	1	Unrelated			
Skin wound	1 (4.2%)	1	Unrelated			
Traumatic ulcer	1 (4.2%)	1	Possibly (1)			
General disorders/procedures						
Impaired healing	1 (4.2%)	1	Unrelated	2 (8.3%)	2	Possibly (1), unrelated (1)
Influenza like illness	3 (12.5%)	6	Unlikely (1), Unrelated (5)			
Cholelithiasis				1 (4.2%)	1	Unrelated
Soft tissue neoplasm				1 (4.2%)	1	Unrelated
Peripheral swelling				1 (4.2%)	1	Unlikely (1)
Scar excision				1 (4.2%)	1	Unrelated
Peripheral coldness	1 (4.2%)	1	Unrelated			
Skin and subcutaneous tissue disorders						
Excessive granulation tissue	2 (8.3%)	2	Possibly (1), unrelated (1)			
Pruritus	2 (8.3%)	2	Unlikely (1), possibly (1)			
Other^a^	5 (21%)	6	Unlikely (3), unrelated (3)			
Nervous system disorders						
Paresthesia	1 (4.2%)	1	Unrelated			
Meralgia paraesthetica				1 (4.2%)	1	Unrelated
Nerve compression				1 (4.2%)	1	Unrelated

Total safety period is defined as the time between first biopsy procedure and end of the study.

The bold text gives overall safety data, which are further specified in the rest of the table.

dS: denovoSkin^™^; n: number of patients; nae: number of adverse events;

a Alopecia, eczema, skin pain, scar pain, skin swelling.

b Serious adverse events are a subset of total adverse events.

**Figure 4.**
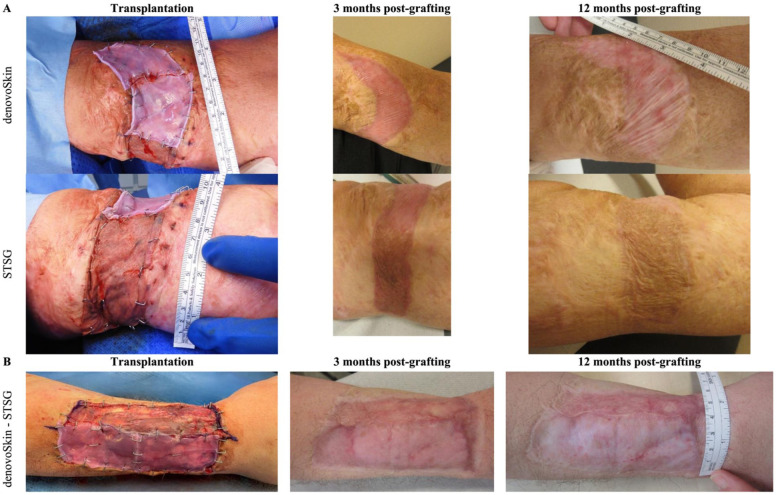
Longitudinal clinical follow-up of denovoSkin™ versus STSG in two patient requiring scar revision: (a) upper arm, scar incision and (b) forearm, scar excision.

Longitudinal clinical follow-up images of two patients are presented in Figure 4. Corresponding video documentation is available at 3 months for patient 1 (Supplemental Video, Supplemental Digital Content 1) and at 12 months for patient 2 (Supplemental Video, Supplemental Digital Content 2).

### Primary outcome – POSAS observer score at 3 months

**Figure 5.**
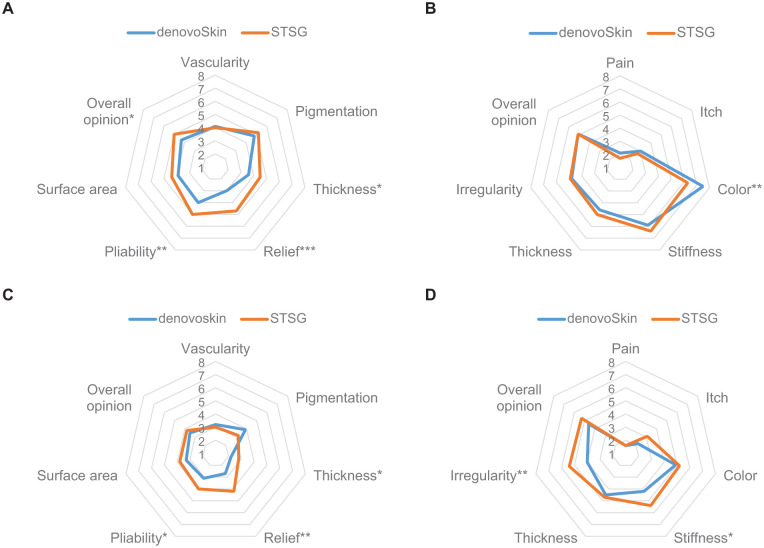
Mean POSAS observer (a and c) and patient (b and d) scores at 3 (upper) and 12 months (lower) post-grafting. Symbols indicate statistically significant differences between denovoSkin^™^ and STSG. *p < 0.05. **p < 0.01. ***p < 0.001.

A visual presentation of observer-rated scar quality items at 3 months is shown in Figure 5(a). At 3 months post-grafting, the mean observer *total* score was 23.4 (SD 10.0) for denovoSkin™ areas versus 27.9 (SD 9.5) for STSG areas (*p* = 0.008). In half of the cases (11 out of 21, 52%), denovoSkin™ areas were hypopigmented compared to normal skin. DenovoSkin™ significantly outperformed STSG for *thickness* (*p* = 0.031), *relief* (*p* < 0.001), *pliability* (*p* = 0.006), and *overall opinion* (*p* = 0.017) at 3 months. The detailed POSAS observer data at 3, 6, and 12 months post-burn are provided in Supplemental Table 1.

### Other scar quality outcomes

#### POSAS observer score at 12 months

A visual comparison of observer-rated scar quality items at 12 months is shown in Figure 5(c). The mean observer *total* score was 18.5 (±7.6) for denovoSkin™ areas at 12 months post-grafting, versus 20.9 (±5.3) for STSG (*p* = 0.093). Significant differences in favor of denovoSkin™ were observed at 12 months for *thickness* (*p* = 0.030), *relief* (*p* = 0.002), and *pliability* (*p* = 0.049).

#### POSAS patient score

The detailed POSAS patient data at 3, 6, and 12 months post-burn are provided in Supplemental Table 2. A visual presentation of patient-rated scar quality items at 3 and 12 months is shown in Figure 5(b) and (d), respectively. At 3 months, the *overall opinion on scar quality* of both areas was rated as similar by patients (5.1 ± 2.9 for denovoSkin™ and 5.1 ± 2.5 for STSG). *Color* significantly differed between denovoSkin™ and STSG at 3 months (7.5 ± 1.9 vs 6.3 ± 2.8, *p* = 0.005). Patients preferred denovoSkin™ for stiffness (*p* < 0.05) and irregularity (*p* < 0.01).

#### Cutometer

**Figure 6.**
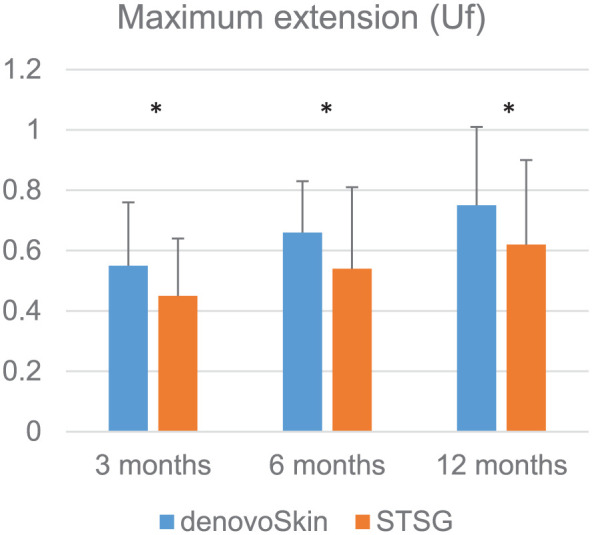
Mean cutometer values (maximum extension (Uf)) in 20 patients for denovoSkin™ (blue) and STSG (orange) versus the values for the respective normal skin at 3, 6, and 12 months post-grafting. Symbol indicates a statistically significant difference between denovoSkin^™^ and STSG. *p < 0.05.

Figure 6 shows maximum extension (Uf) values for denovoSkin™ and STSG at 3, 6, and 12 months post-grafting. The detailed Cutometer data are provided in Supplemental Table 3. Maximum extension (Uf) values were significantly better for denovoSkin™ at 3, 6, and 12 months post-grafting (*p* < 0.04).

#### Colormeter

The detailed Colormeter data are provided in Supplemental Table 4. No significant differences between the two areas were found at 3, 6, and 12 months post-grafting.

## Discussion

We evaluated the safety and efficacy of the novel bio-engineered skin graft denovoSkin™ in patients requiring coverage of full thickness skin defects. DenovoSkin™ demonstrated statistically significant superior scar quality compared to STSG at 3 months, which was maintained at six and 12 months. In addition, denovoSkin™ achieved a markedly greater donor-site-to-surface ratio. No significant safety issues were encountered. These findings confirm that denovoSkin™ provides durable, high-quality skin coverage while minimizing donor site.

In reconstructive practice, FTSGs generally provide the most favorable functional and esthetic results; however, their use is severely limited by donor-site availability. DenovoSkin™ overcomes this limitation by offering skin coverage of full-thickness skin quality from a minimal biopsy. Thereby, denovoSkin™ provides stable coverage and significantly reduces donor-site morbidity – a major advantage for patients with extensive skin defects or those requiring multiple staged reconstructions. Preliminary observations in pediatric patients also suggest that denovoSkin™ may expand with growth, potentially reducing the need for secondary procedures later in life.

Over the past decades, several autologous bi-layered constructs containing fibroblasts and keratinocytes have been developed to improve outcomes in extensive wounds, such asself-assembled skin substitutes (SASSs) and engineered skin substitutes (ESSs). SASSs are generated by allowing fibroblasts to produce their own extracellular matrix in culture, onto which keratinocytes are subsequently seeded, whereas ESSs are composed of autologous fibroblasts and keratinocytes seeded on a biodegradable scaffold.^24,25^ Both have shown promising results in acute burn treatment, demonstrating stable wound closure, and reduced donor-site requirements in severely burned patients.^24,26,27^ However, such constructs have not yet been evaluated in controlled trials for reconstructive indications. Like these full skin constructs, denovoSkin™ contains autologous viable epidermal and dermal cells, which together with the compressed collagen component provides the essential structure for skin regeneration rather than scar formation. After a successful phase I study, a phase II study in adult and pediatric burn patients, and several compassionate use cases in acute burn treatment,^18–21^ the current study is the first to evaluate the safety and efficacy of denovoSkin™ on improving scar quality in reconstructive surgery.

Pigmentation remains a challenge in scars. The limited presence of melanocytes in the current composition of denovoSkin™ is reflected by the scores on the item *pigmentation* in the POSAS observer scale. DenovoSkin™ relies on peripheral pigment cell repopulation during graft maturation,^21^ which explains the hypopigmented appearance of denovoSkin™. Nonetheless, we observed improvement of pigmentation over time, and re-introduction of melanocytes could be achieved by treatment of the hypopigmented areas using melanocyte spray techniques.^28^ Preclinical developments have shown the feasibility of introducing melanocytes and even pre-vascularized structures in bioengineered skin constructs.^15,22,29,30^ Additionally, future developments might also extend to regeneration of hair follicles, sebaceous glands, and sweat glands.^31,32^ Such innovations are expected to further enhance functional and esthetic outcomes of bio-engineered skin constructs. An often-mentioned limitation of tissue-engineered skin is the manufacturing time. While relevant in acute settings, it is generally not an issue in reconstructive surgery. The biopsy-to-grafting interval is approximately 4 weeks, allowing for planned interventions, and cryopreservation of the autologous cells could even enable future re-use or delayed applications.

This study addresses several strengths and limitations. The intra-patient design allowed for direct comparison of the two treatment modalities under nearly identical wound environments, minimizing inter-subject variability. The long-term follow up and quantitative outcome evaluations in a multicenter trial setup are also strengths of this study, since these provide statistical power to the outcome analysis. This study also has some limitations. First, the sample size was relatively small, and although powered for the primary endpoint, it may not have been sufficient to detect smaller differences in secondary outcomes. Second, wound healing progression was assessed at predefined visits, therefore, progress between visits—for example, between day 28 and two months—may have gone unnoted, leading to the assessment of longer healing times. Nevertheless, the somewhat slower epithelialization of denovoSkin™ is likely due to the fact that it is produced submerged. Therefore, the epithelial differentiation is incomplete when applied to the wound and needs to be completed in situ after grafting (see Figure 2 for a histological image of the product prior to transplantation). In contrast, STSGs are already fully differentiated, and re-epithelialization occurs through cell migration from the edge of the meshes, resulting in faster wound closure. Also, denovoSkin™ requires neovascularization of the dermal component before full epithelization can be completed, similar to dermal templates.^33^

Finally, one may question whether comparing denovoSkin™ to STSG represents the most appropriate comparison. FTSGs may more closely resemble the structure of denovoSkin™, however, the clinical applicability of FTSGs is limited due to high donor site constraints, especially in patients with large skin defects. Although the defects treated in this clinical trial were relatively modest in size, future use of denovoSkin™ in reconstruction would allow coverage of much larger defects. Also, STSGs remain commonly used, especially in burn reconstructive settings, where there is a limitation in donor sites for other reconstructive techniques, making these a relevant comparator in this trial.

## Conclusion

This study demonstrates that denovoSkin™ is a safe and effective treatment modality for the reconstruction of full-thickness skin defects. Its ability to provide autologous, permanent, and full-thickness skin coverage makes it suitable for a broad range of indications, such as large full-thickness wounds, post-oncologic defects, contracture releases, traumatic injuries and chronic wound management. Significant advantages in scar quality—particularly in thickness, relief, pliability, elasticity and surface regularity—suggest that tissue-engineered skin substitutes may play a growing role in future reconstructive strategies.

## Supplemental Material

sj-docx-1-tej-10.1177_20417314261429663 – Supplemental material for Safety and efficacy of bio-engineered, autologous dermo-epidermal skin grafts in reconstructive surgery: 1-year results of a prospective, randomized, intra-patient controlled, multicenter phase II clinical trial Supplemental material, sj-docx-1-tej-10.1177_20417314261429663 for Safety and efficacy of bio-engineered, autologous dermo-epidermal skin grafts in reconstructive surgery: 1-year results of a prospective, randomized, intra-patient controlled, multicenter phase II clinical trial by Frederique M. Kemme, Anouk Pijpe, Matthea M. Stoop, Paul P. M. van Zuijlen, Martin Meuli, Clemens Schiestl, Fabienne Hartmann-Fritsch, Bong-Sung Kim, Kathrin Neuhaus, Jan A. Plock, Daniel Rittirsch, Kathi Mujynya, Ernst Reichmann, Sophie Böttcher-Haberzeth, Melinda Farkas, Jenny Bressan, Marcello Zamparelli, Ilaria Mataro, Carlo Petroccione, Alex Pontini, Bruno Azzena, Daniela Marino and Esther Middelkoop in Journal of Tissue Engineering

sj-docx-2-tej-10.1177_20417314261429663 – Supplemental material for Safety and efficacy of bio-engineered, autologous dermo-epidermal skin grafts in reconstructive surgery: 1-year results of a prospective, randomized, intra-patient controlled, multicenter phase II clinical trial Supplemental material, sj-docx-2-tej-10.1177_20417314261429663 for Safety and efficacy of bio-engineered, autologous dermo-epidermal skin grafts in reconstructive surgery: 1-year results of a prospective, randomized, intra-patient controlled, multicenter phase II clinical trial by Frederique M. Kemme, Anouk Pijpe, Matthea M. Stoop, Paul P. M. van Zuijlen, Martin Meuli, Clemens Schiestl, Fabienne Hartmann-Fritsch, Bong-Sung Kim, Kathrin Neuhaus, Jan A. Plock, Daniel Rittirsch, Kathi Mujynya, Ernst Reichmann, Sophie Böttcher-Haberzeth, Melinda Farkas, Jenny Bressan, Marcello Zamparelli, Ilaria Mataro, Carlo Petroccione, Alex Pontini, Bruno Azzena, Daniela Marino and Esther Middelkoop in Journal of Tissue Engineering

sj-docx-3-tej-10.1177_20417314261429663 – Supplemental material for Safety and efficacy of bio-engineered, autologous dermo-epidermal skin grafts in reconstructive surgery: 1-year results of a prospective, randomized, intra-patient controlled, multicenter phase II clinical trial Supplemental material, sj-docx-3-tej-10.1177_20417314261429663 for Safety and efficacy of bio-engineered, autologous dermo-epidermal skin grafts in reconstructive surgery: 1-year results of a prospective, randomized, intra-patient controlled, multicenter phase II clinical trial by Frederique M. Kemme, Anouk Pijpe, Matthea M. Stoop, Paul P. M. van Zuijlen, Martin Meuli, Clemens Schiestl, Fabienne Hartmann-Fritsch, Bong-Sung Kim, Kathrin Neuhaus, Jan A. Plock, Daniel Rittirsch, Kathi Mujynya, Ernst Reichmann, Sophie Böttcher-Haberzeth, Melinda Farkas, Jenny Bressan, Marcello Zamparelli, Ilaria Mataro, Carlo Petroccione, Alex Pontini, Bruno Azzena, Daniela Marino and Esther Middelkoop in Journal of Tissue Engineering

sj-docx-4-tej-10.1177_20417314261429663 – Supplemental material for Safety and efficacy of bio-engineered, autologous dermo-epidermal skin grafts in reconstructive surgery: 1-year results of a prospective, randomized, intra-patient controlled, multicenter phase II clinical trial Supplemental material, sj-docx-4-tej-10.1177_20417314261429663 for Safety and efficacy of bio-engineered, autologous dermo-epidermal skin grafts in reconstructive surgery: 1-year results of a prospective, randomized, intra-patient controlled, multicenter phase II clinical trial by Frederique M. Kemme, Anouk Pijpe, Matthea M. Stoop, Paul P. M. van Zuijlen, Martin Meuli, Clemens Schiestl, Fabienne Hartmann-Fritsch, Bong-Sung Kim, Kathrin Neuhaus, Jan A. Plock, Daniel Rittirsch, Kathi Mujynya, Ernst Reichmann, Sophie Böttcher-Haberzeth, Melinda Farkas, Jenny Bressan, Marcello Zamparelli, Ilaria Mataro, Carlo Petroccione, Alex Pontini, Bruno Azzena, Daniela Marino and Esther Middelkoop in Journal of Tissue Engineering
