# Supplementary material for: Safety and efficacy of bio-engineered, autologous dermo-epidermal skin grafts in reconstructive surgery: 1-year results of a prospective, randomized, intra-patient controlled, multicenter phase II clinical trial
Source: J Tissue Eng. 2026 Mar 23;17:20417314261429663. doi: 10.1177/20417314261429663 (PMC13013987; doi:10.1177/20417314261429663)
Supplement: sj-docx-1-tej-10.1177_20417314261429663 – Supplemental material for Safety and efficacy of bio-engineered, autologous dermo-epidermal skin grafts in reconstructive surgery: 1-year results of a prospective, randomized, intra-patient controlled, multicenter phase II clinical trial [file sj-docx-1-tej-10.1177_20417314261429663.docx]

**Supplementary Table 1**. Mean POSAS **observer** scores at 3, 6 and 12 months post-grafting*

| POSAS item | 3 months  N=21 | | | | | | 6 months  N=-21 | | | | | | 12 months  N=21 | | | | | |
| --- | --- | --- | --- | --- | --- | --- | --- | --- | --- | --- | --- | --- | --- | --- | --- | --- | --- | --- |
|  | **denovoSkin** | | **STSG** | |  |  | **denovoSkin** | | **STSG** | |  |  | **denovoSkin** | | **STSG** | |  |  |
|  | **Mean** | **SD** | **Mean** | **SD** | **p value** | **mean diff (SD)** | **Mean** | **SD** | **Mean** | **SD** | **p value** | **mean diff (SD)** | **Mean** | **SD** | **Mean** | **SD** | **p value** | **mean diff (SD)** |
| Vascularity | 4.1 | 1.5 | 4.0 | 1.8 | 0.679 | 0.1 (1.6) | 3.5 | 1.4 | 3.1 | 1.4 | 0.057 | 0.4 (0.9) | 3.2 | 1.3 | 3.0 | 1.3 | 0.329 | 0.3 (1.3) |
| Pigmentation | 4.8 | 2.0 | 5.2 | 1.9 | 0.196 | -0.5 (1.6) | 4.5 | 1.3 | 3.7 | 1.0 | **0.035** | 0.9 (1.7) | 3.9 | 1.6 | 3.2 | 1.1 | 0.090 | 0.7 (1.7) |
| Thickness | 3.6 | 1.9 | 4.5 | 1.9 | **0.031** | -0.9 (1.8) | 2.8 | 1.5 | 3.1 | 1.4 | 0.258 | -0.4 (1.5) | 2.2 | 1.3 | 2.8 | 1.4 | **0.030** | -0.5 (1.0) |
| Relief | 3.0 | 1.7 | 4.7 | 2.0 | **<0.001** | -1.7 (1.7) | 3.1 | 1.6 | 4.1 | 1.3 | **0.046** | -1.1 (2.4) | 2.7 | 1.6 | 4.2 | 1.1 | **0.002** | -1.5 (1.9) |
| Pliability | 4.0 | 2.3 | 5.0 | 2.0 | **0.006** | -1.15 (1.6) | 3.0 | 1.7 | 4.0 | 1.4 | 0.051 | -1.1 (2.3) | 3.1 | 1.7 | 4.0 | 1.5 | **0.049** | -0.9 (1.9) |
| Surface area | 3.9 | 2.4 | 4.4 | 1.7 | 0.134 | -0.5 (1.5) | 3.1 | 1.5 | 3.5 | 1.7 | 0.305 | -0.4 (1.7) | 3.3 | 2.3 | 3.8 | 2.2 | 0.196 | -0.5 (1.6) |
| Overall opinion | 4.3 | 1.6 | 5.0 | 1.6 | **0.017** | -0.8 (1.3) | 3.5 | 1.2 | 4.0 | 0.9 | 0.083 | -0.4 (1.1) | 3.5 | 1.2 | 3.8 | 0.9 | 0.300 | -0.3 (1.2) |
| Total | 23.4 | 10.0 | 27.9 | 9.5 | **0.008** | -4.5 (7.0) | 19.9 | 6.4 | 21.6 | 5.2 | 0.232 | -1.7 (6.2) | 18.5 | 7.6 | 20.9 | 5.3 | 0.093 | -2.4 (6.2) |

**Higher scores indicate greater deviation from uninjured skin.*

*Mean difference = denovoSkin – STSG*
