# Supplementary material for: Safety and efficacy of bio-engineered, autologous dermo-epidermal skin grafts in reconstructive surgery: 1-year results of a prospective, randomized, intra-patient controlled, multicenter phase II clinical trial
Source: J Tissue Eng. 2026 Mar 23;17:20417314261429663. doi: 10.1177/20417314261429663 (PMC13013987; doi:10.1177/20417314261429663)
Supplement: sj-docx-2-tej-10.1177_20417314261429663 – Supplemental material for Safety and efficacy of bio-engineered, autologous dermo-epidermal skin grafts in reconstructive surgery: 1-year results of a prospective, randomized, intra-patient controlled, multicenter phase II clinical trial [file sj-docx-2-tej-10.1177_20417314261429663.docx]

**Supplementary Table 2.** Mean POSAS **patient** scores at 3, 6 and 12 months post-grafting*

| POSAS item | 3 months  N=21 | | | | | | 6 months  N=21 | | | | | | 12 months  N=21 | | | | | |
| --- | --- | --- | --- | --- | --- | --- | --- | --- | --- | --- | --- | --- | --- | --- | --- | --- | --- | --- |
|  | **denovoSkin** | | **STSG** | |  |  | **denovoSkin** | | **STSG** | |  |  | **denovoSkin** | | **STSG** | |  |  |
|  | **Mean** | **SD** | **Mean** | **SD** | **p value** | **mean diff (SD)** | **Mean** | **SD** | **Mean** | **SD** | **p value** | **mean diff (SD)** | **Mean** | **SD** | **Mean** | **SD** | **p value** | **mean diff (SD)** |
| Pain | 2.1 | 2.3 | 1.7 | 1.3 | 0.336 | 0.5 (2.0) | 2.0 | 2.1 | 1.9 | 1.7 | 0.806 | 0.1 (1.8) | 1.6 | 1.5 | 1.6 | 1.2 | 0.890 | 0.1 (1.6) |
| Itch | 3.0 | 2.8 | 2.7 | 2.2 | 0.453 | 0.5 (2.6) | 3.3 | 2.9 | 3.9 | 3.6 | 0.131 | -0.6 (1.8) | 2.2 | 2.0 | 3.1 | 3.0 | 0.055 | -0.9 (1.9) |
| Color | 7.5 | 1.9 | 6.3 | 2.8 | **0.005** | 1.2 (1.8) | 6.3 | 2.5 | 5.6 | 3.0 | 0.224 | 0.7 (2.6) | 4.9 | 1.9 | 5.2 | 2.7 | 0.491 | -0.3 (2.2) |
| Stiffness | 5.9 | 3.0 | 6.4 | 2.5 | 0.411 | -0.5 (2.9) | 5.2 | 2.9 | 5.6 | 3.1 | 0.566 | -0.4 (3.0) | 4.2 | 2.4 | 5.4 | 2.4 | **0.046** | -1.2 (2.6) |
| Thickness | 4.6 | 2.9 | 5.0 | 3.2 | 0.623 | -0.4 (3.5) | 5.2 | 3.3 | 5.4 | 3.4 | 0.753 | -0.2 (2.7) | 4.5 | 2.2 | 4.7 | 2.8 | 0.827 | -0.1 (2.0) |
| Irregularity | 4.8 | 2.9 | 4.9 | 3.0 | 0.913 | -0.1 (3.9) | 4.5 | 3.2 | 6.2 | 3.2 | **0.012** | -1.7 (2.8) | 4.1 | 2.5 | 5.4 | 2.7 | **0.008** | -1.4 (2.2) |
| Overall opinion | 5.1 | 2.9 | 5.1 | 2.5 | 1.000 | 0.0 (3.2) | 4.7 | 2.6 | 5.5 | 3.0 | 0.115 | -0.8 (2.1) | 4.6 | 2.0 | 5.3 | 2.6 | 0.235 | -0.7 (2.5) |
| Total | 27.9 | 12.2 | 26.71 | 11.1 | 0.654 | 1.2 (12.0) | 26.4 | 13.7 | 28.5 | 15.1 | 0.333 | -2.1 (9.7) | 21.5 | 9.5 | 25.1 | 13.0 | 0.096 | -3.7 (9.6) |

**Higher scores indicate greater deviation from uninjured skin.*

*Mean difference = denovoSkin – STSG*
