# Supplementary material for: Safety and efficacy of bio-engineered, autologous dermo-epidermal skin grafts in reconstructive surgery: 1-year results of a prospective, randomized, intra-patient controlled, multicenter phase II clinical trial
Source: J Tissue Eng. 2026 Mar 23;17:20417314261429663. doi: 10.1177/20417314261429663 (PMC13013987; doi:10.1177/20417314261429663)
Supplement: sj-docx-3-tej-10.1177_20417314261429663 – Supplemental material for Safety and efficacy of bio-engineered, autologous dermo-epidermal skin grafts in reconstructive surgery: 1-year results of a prospective, randomized, intra-patient controlled, multicenter phase II clinical trial [file sj-docx-3-tej-10.1177_20417314261429663.docx]

**Supplementary Table 3.** Mean Cutometer values* at 3, 6, and 12 months post-grafting**

|  | 3 months  N=20 | | | | | 6 months  N=20 | | | | | 12 months  N=20 | | | | | |
| --- | --- | --- | --- | --- | --- | --- | --- | --- | --- | --- | --- | --- | --- | --- | --- | --- |
|  | **denovoSkin** | | **STSG** | |  | **denovoSkin** | | **STSG** | |  | **denovoSkin** | | | **STSG** | |  |
|  | **Mean** | **SD** | **Mean** | **SD** | **p value** | **Mean** | **SD** | **Mean** | **SD** | **p value** | **Mean** | **SD** | **Mean** | | **SD** | **p value** |
| Maximum Extension (Uf) | 0.55 | 0.21 | 0.45 | 0.19 | **0.035** | 0.66 | 0.17 | 0.54 | 0.27 | **0.040** | 0.75 | 0.26 | 0.62 | | 0.28 | **0.021** |

**Presented as the ratio versus uninjured skin.*

**R0 values equal to 0 were excluded from the analysis. The number of excluded values (out of 5 measurements per treatment area) per time point was: 3 months: 9/254 (3.5%); 6 months; 3/256 (1.2%); 12 months: 6/274 (2.2%).
