# Supplementary material for: Safety and efficacy of bio-engineered, autologous dermo-epidermal skin grafts in reconstructive surgery: 1-year results of a prospective, randomized, intra-patient controlled, multicenter phase II clinical trial
Source: J Tissue Eng. 2026 Mar 23;17:20417314261429663. doi: 10.1177/20417314261429663 (PMC13013987; doi:10.1177/20417314261429663)
Supplement: sj-docx-4-tej-10.1177_20417314261429663 – Supplemental material for Safety and efficacy of bio-engineered, autologous dermo-epidermal skin grafts in reconstructive surgery: 1-year results of a prospective, randomized, intra-patient controlled, multicenter phase II clinical trial [file sj-docx-4-tej-10.1177_20417314261429663.docx]

**Supplementary Table 4.** Mean Colormeter values* at 3, 6, and 12 months post-grafting

|  | 3 months  N=18 | | | | | 6 months  N=21 | | | | | 12 months  N=21 | | | | |
| --- | --- | --- | --- | --- | --- | --- | --- | --- | --- | --- | --- | --- | --- | --- | --- |
|  | **denovoSkin** | | **STSG** | |  | **denovoSkin** | | **STSG** | |  | **denovoSkin** | | **STSG** | |  |
|  | **Mean** | **SD** | **Mean** | **SD** | **p value** | **Mean** | **SD** | **Mean** | **SD** | **p value** | **Mean** | **SD** | **Mean** | **SD** | **p value** |
| Erythema | 5.44 | 1.96 | 5.59 | 3.31 | 0.82 | 4.66 | 2.76 | 4.26 | 2.44 | 0.41 | 3.22 | 1.97 | 3.15 | 2.91 | 0.92 |
| Melanin | 14.60 | 6.71 | 13.84 | 7.36 | 0.55 | 7.08 | 6.33 | 9.03 | 4.70 | 0.14 | 5.64 | 4.61 | 7.68 | 4.91 | 0.11 |

**Presented as the difference with uninjured skin.*
